# Indoxyl Sulfate Might Play a Role in Sarcopenia, While Myostatin Is an Indicator of Muscle Mass in Patients with Chronic Kidney Disease: Analysis from the RECOVERY Study

**DOI:** 10.3390/toxins14100660

**Published:** 2022-09-23

**Authors:** Su Mi Lee, Mi Yeun Han, Su Hyun Kim, Ran Hui Cha, Seock Hui Kang, Jun Chul Kim, Won Suk An

**Affiliations:** 1 Department of Internal Medicine, Dong-A University College of Medicine, Busan 49201, Korea; 2 Department of Internal Medicine, Hallym University Hangang Sacred Heart Hospital, Seoul 07247, Korea; 3 Department of Internal Medicine, Chung-Ang University Gwangmyeong Hospital, Chung-Ang University College of Medicine, Gwangmyeong 14353, Korea; 4 Department of Internal Medicine, National Medical Center, Seoul 04564, Korea; 5 Department of Internal Medicine, Yeungnam University College of Medicine, Daegu 42415, Korea; 6 Department of Internal Medicine, CHA Gumi Medical Center, CHA University, Gumi 39295, Korea

**Keywords:** chronic kidney disease, indoxyl sulfate, myostatin, sarcopenia

## Abstract

Serum myostatin and indoxyl sulfate (IS) levels increase with kidney function decline and may function as uremic toxins in chronic kidney disease (CKD)-related sarcopenia. Herein, we analyzed the association between serum myostatin and IS levels and sarcopenia in patients with CKD, by performing a post hoc analysis of baseline data extracted from the RECOVERY study (clinicaltrials.gov: NCT03788252) of 150 patients with CKD. We stratified patients into two groups according to the median value of myostatin (cutoff 4.5 ng/mL) and IS levels (cutoff 0.365 mg/dL). The proportion of patients with sarcopenia was higher in those with high IS levels but lower in those with high myostatin levels. The skeletal muscle mass index (SMI) and handgrip strength (HGS) were significantly lower in patients with high IS levels but significantly higher in patients with high myostatin levels. IS levels showed a negative correlation with glomerular filtration rate (GFR), SMI, and HGS. However, myostatin levels were positively correlated with SMI and HGS, but not with GFR. Sarcopenia was independently associated with age and IS level after adjustment. Increased levels of serum total IS might play a role in sarcopenia, while increased levels of serum myostatin are associated with muscle mass in patients with CKD.

## 1. Introduction

According to the National Health and Nutrition Survey in Korea, the prevalence of chronic kidney disease (CKD) in adults aged > 30 years in Korea was 9.3% in 2019, indicating an over three-fold increase from 2.5% in 2009. Sarcopenia is a condition characterized by concomitant loss of skeletal muscle mass, strength, and function. Sarcopenia is related to pathologic conditions as well as aging, and CKD is a known cause of sarcopenia. Sarcopenia is common in CKD and is associated with adverse clinical outcomes, such as cardiovascular complications and the risk of death [1,2]. Consequently, there is a need for the early identification and prevention of sarcopenia in patients with CKD.

Myostatin, a member of the transforming growth factor-β family, acts as a negative regulator of skeletal muscle growth. Myostatin suppresses skeletal muscle growth, and myostatin inhibition leads to muscle hypertrophy. Indoxyl sulfate (IS), also known as 3-indoxylsulfate and 3-indoxylsulfuric acid, is a metabolite of dietary L-tryptophan in the intestine and functions as a uremic toxin. In a CKD animal model, the presence of IS could increase myostatin expression potentially driving skeletal muscle atrophy [3]; however, a recent study did not find the same results [4]. Myostatin and IS levels increase with a decrease in kidney function [5,6]. Therefore, myostatin and IS may function as important uremic toxins related to sarcopenia in patients with CKD. However, a recent report showed that myostatin levels increased in patients with CKD who were balance-trained. In addition, myostatin levels were associated with increased lean mass [7]. This study aimed to assess the association between serum myostatin and IS levels and sarcopenia in patients with CKD.

## 2. Results

### 2.1. Baseline Characteristics

A total of 150 participants with a mean age of 65.0 ± 10.8 years were enrolled in the study (Table S1). Among the participants, 64.7% were male and 50.6% had diabetes. The mean serum creatinine levels were 2.1 ± 0.7 mg/dL, and estimated glomerular filtration rate (eGFR) levels were 33.8 ± 12.5 mL/min/1.73 m^2^. Overall, 37 (24.8%) patients had CKD stage 3A, 51 (34.0%) had CKD stage 3B, 54 (36.0%) had CKD stage 4, and eight (5.3%) had CKD stage 5 (Table S2). The mean serum myostatin levels were 4.8 ± 2.0 ng/mL, and IS levels were 0.5 ± 0.4 mg/dL.

### 2.2. Comparison of Characteristics According to Myostatin Levels

We classified the patients into two groups according to the median value of myostatin: patients with high myostatin levels (≥4.5 pg/mL) and those with low myostatin levels (<4.5 pg/mL) (Table 1). Higher handgrip strength (HGS), skeletal muscle mass index (SMI), body mass index, and myostatin/SMI ratio, and male sex were more common in patients with high myostatin levels than in those with low myostatin levels. SMI and HGS were significantly higher (8.1 ± 1.1 kg/m^2^ vs. 7.3 ±1.2 kg/m^2^, *p* < 0.001 and 30.6 ± 7.7 kg vs. 26.2 ± 9.6 kg, *p* = 0.003) in patients with high myostatin levels than in those with low myostatin levels. The proportion of patients with presarcopenia and sarcopenia was significantly lower in those with high myostatin levels than in those with low myostatin levels. The levels of eGFR, 25-hydroxyvitamin D [25(OH)D], C-reactive protein (CRP), tumor necrosis factor-alpha (TNFα), interleukin-6 (IL-6), and IS showed no difference between the groups.

Correlation analysis was performed to determine the association between myostatin levels and variables (Table 2 and Table S3). Myostatin levels were negatively associated with IS and positively associated with HGS and SMI but were not correlated with creatinine or eGFR. The myostatin/SMI ratio was positively correlated with creatinine (r = 0.188, *p* = 0.022) and negatively correlated with eGFR (r = −0.167, *p* = 0.043); however, it was not correlated with SMI or HGS (r = −0.011, *p* = 0.896 and r = 0.072, *p* = 0.383).

### 2.3. Comparison of Characteristics According to IS Levels

We further classified the patients into two groups according to the median IS value: patients with high IS levels (≥0.365 mg/dL) and those with low IS levels (<0.365 mg/dL) (Table 3). Lower HGS, SMI, and eGFR, and female sex were more prevalent in patients with high IS levels than in those with low IS levels. SMI and HGS were significantly lower (7.3 ± 1.2 kg/m^2^ vs. 8.2 ± 1.1 kg/m^2^, *p* < 0.001 and 26.0 ± 8.2 kg vs. 30.9 ± 9.2 kg, *p* = 0.001) in patients with high IS levels than in those with low IS levels. The proportion of patients with presarcopenia and sarcopenia was significantly higher in those with high IS levels than in those with low IS levels. The levels of CRP, TNFα, myostatin, and myostatin/SMI ratio did not differ between the groups.

Correlation analysis was performed to determine the association between IS levels and variables (Table 2). IS levels were found to be negatively associated with eGFR, HGS, and SMI. In addition, IS levels were positively associated with creatinine levels.

### 2.4. Factors Associated with Presarcopenia and Sarcopenia

We subsequently performed multiple logistic regression analysis to clarify the factors associated with presarcopenia and sarcopenia (Table 4). Presarcopenia and sarcopenia were independently associated with age and IS levels after adjusting for sex, diabetes mellitus, creatinine level, and myostatin/SMI.

**Figure 1:**
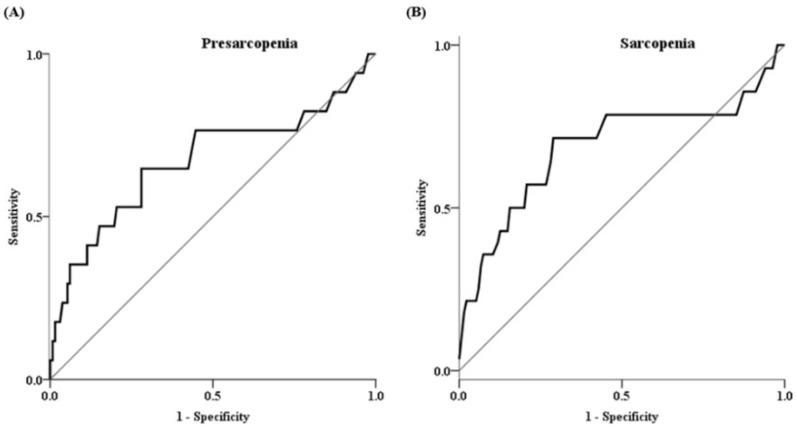
Receiver operating characteristic curve of IS levels for predicting the probability of presarcopenia (**A**) and sarcopenia (**B**).

We further estimated the receiver operator characteristic (ROC) curve for the diagnosis of presarcopenia and sarcopenia using the IS levels (Figure 1). The areas under the curves (AUCs) for presarcopenia and sarcopenia were 0.67 (95% confidence interval [1], 0.51–0.84; *p* = 0.022) and 0.69 (95% CI, 0.51–0.87; *p* = 0.021), respectively. The sensitivity for predicting presarcopenia and sarcopenia were 64.7% and 71.4%, respectively, with specificity of 64.4% and 71.9%, respectively.

## 3. Discussion

IS is a well-known protein-bound uremic toxin that is difficult to remove with dialysis, and IS levels increase as kidney function decreases. IS is associated with adverse outcomes in patients with CKD, including cardiovascular complications and mortality [8]. Furthermore, recent in vitro and in vivo studies have reported that IS accumulates in muscle cells through organic anion transporters. The accumulation of IS leads to the production of myostatin and atrogen-1 through activation of oxidative stress and incremental increases of inflammatory cytokines. Finally, sarcopenia may be induced by mitochondrial dysfunction of muscle fibers [1]. In our study, IS levels were negatively correlated with SMI and HGS levels. High IS levels are an independent risk factor for sarcopenia and presarcopenia in CKD. IS levels were positively correlated with creatinine and negatively correlated with eGFR using the CKD Epidemiology Collaboration equations, confirming that IS is a uremic toxin. Therefore, monitoring or reducing IS levels is promising approach in delaying sarcopenia. Further research is necessary to elucidate on the effect of reducing IS levels in the genesis or progression of sarcopenia.

Myostatin is predominantly produced in skeletal muscles, where it functions to suppress skeletal muscle growth. Myostatin levels are increased in patients with CKD, and increased myostatin may induce inflammatory changes and mitochondrial dysfunction of muscle fibers in patients with CKD [9]. Therefore, increased myostatin levels may be related to sarcopenia. However, the relationship between serum myostatin levels and muscle mass remains uncertain, with conflicting results. Muscle hypertrophy was observed in myostatin knockout mice [10] and myostatin administration induced cachexia in mice [11]. Muscle hypertrophy has further been observed in children with myostatin mutations [12] and an inverse association between serum myostatin levels and muscle mass has been identified in patients with chronic obstructive pulmonary disease and heart failure [13,14]. Decreasing myostatin levels without inducing changes in muscle mass by exercise or high-flux dialysis improves muscle power in patients undergoing hemodialysis [15,16]. However, myostatin levels were increased in patients with CKD who were balance-trained and were associated with increased lean mass. Conversely, higher myostatin levels are associated with higher muscle mass in patients undergoing peritoneal dialysis (PD) [17]. In our study, we found that higher serum myostatin levels were associated with higher muscle mass and positively associated with HGS and SMI in patients with CKD. Similar data between muscle mass and myostatin levels have been previously reported in patients undergoing dialysis [17,18]. It can be assumed that myostatin production is reduced in patients with CKD with lower muscle mass. In other words, the low myostatin level reflects a low proportion of intact muscle mass in patients with CKD. It can be presumed that the higher the myostatin level, the greater the muscle mass in CKD. An increase in myostatin in skeletal muscle and serum occurred after resistance training, along with a subsequent incremental change in follistatin-like-related genes and decrement of activin 2b receptor [19]. The exact roles of myostatin and its mechanism related to muscle mass require further study.

Chronic inflammation, dysbiosis, uremia, and low physical activity all induce myostatin activation. However, the relatively fewer intact muscle fibers in patients with CKD may result in decreased myostatin expression and secretion. Therefore, the myostatin/SMI ratio is finally obtained by correcting myostatin levels according to muscle mass, as the myostatin/SMI ratio reflects myostatin levels corrected for muscle mass. In patients with lower muscle mass, a high myostatin/SMI ratio may indicate that the myostatin level corrected for muscle mass is relatively high because myostatin secretion itself is low due to the lower muscle mass. In a previous report, the myostatin/SMI ratio in older Korean women was negatively associated with HGS [7] but the myostatin/SMI ratio was not presented as an indicator of sarcopenia in CKD in this study. The myostatin level was not correlated with eGFR, but the myostatin/SMI ratio was positively correlated with eGFR. Further studies are needed to identify the role of the myostatin/ASM ratio in the prediction of sarcopenia or physical performance in patients with CKD.

Our study suggests that IS levels are more appropriate than myostatin levels to predict sarcopenia. The inverse correlation between myostatin and IS levels was unexpected. We presume that this inverse correlation was observed because the increase in myostatin levels was related to the increase in muscle mass in this study. This is the first report of the association between serum myostatin and IS levels and sarcopenia in patients with CKD. We found that high IS levels were independent risk factors for sarcopenia in CKD, but myostatin levels were not.

This study has several limitations. Firstly, as a post hoc study of the RECOVERY trial, the available data were insufficient to reveal the cause–effect relationship between the different parameters that were studied. Second, information on sarcopenia, including diet, was not collected. Third, because there were no healthy volunteers, the difference between healthy volunteers and patients with CKD could not be identified.

## 4. Conclusions

In conclusion, IS levels are an important predictor of sarcopenia, while serum myostatin levels are not. IS levels are also positively correlated with IL-6 levels and increase with decreased eGFR. IS levels are not only markers reflecting reduced eGFR but also uremic toxins related to sarcopenia. Further studies are needed to understand why patients with CKD differ from the general population in terms of the relationship between myostatin levels and muscle mass. Further studies are needed to identify new markers related to muscle mass and myostatin, such as the myostatin/SMI ratio, in patients with CKD.

## 5. Materials and Methods

### 5.1. Study Design and Patients

This investigation was a post hoc analysis of baseline data extracted from the RECOVERY study [20]. The study design and patient characteristics have been published previously [20,21]. Briefly, the RECOVERY study was a 48-week, randomized controlled, parallel group, open-label, multicenter trial that evaluated the role of AST-120 in sarcopenia prevention in predialysis in patients with CKD. The key inclusion criteria were age ≥ 20 years, predialysis CKD, serum creatinine 2.0–5.0 mg/dL or eGFR 15–60 mL/min per 1.73 m^2^ calculated by the Chronic Kidney Disease Epidemiology Collaboration Equation [22], serum albumin ≥ 3.0 g/dL, no previous history of taking AST-120 in 4 weeks, able to ambulate with or without assistive devices, and willingness to provide informed consent. Key exclusion criteria included passage disorders in the gastrointestinal tract and uncontrolled constipation, history of kidney transplantation, use of immunosuppressant agents, uncontrolled hypertension (systolic blood pressure ≥ 180 mmHg and diastolic blood pressure ≥ 110 mmHg), acute coronary syndrome within three months, acute infectious or inflammatory illness, progressive malignancy, pregnancy, lactation, or planning to become pregnant during the study period.

A total of 150 patients were recruited, one of whom was excluded because of a lack of data. Ultimately, 149 patients were included in the final analysis. Informed consent was obtained from all the enrolled patients. This study was approved by the Dong-A Institutional Review Board (IRB No. 18-182) and was conducted in accordance with the principles of the Declaration of Helsinki.

### 5.2. Laboratory Data

Blood samples were obtained, processed, refrigerated, and stored at −70 °C until analysis at the central laboratory institution (Seoul Clinical Laboratories, Yongin-si, Gyeonggi-do, Korea). The analytical methods have been published previously [20,21]. Briefly, myostatin, IL-6, and TNFα levels were measured by enzyme-linked immunosorbent assay (ELISA) using DGDF80 (GDF-8/Myostatin Quantikine ELISA Kit, R&D Systems), HS600C (Human IL-6 Quantikine HS ELISA Kit), and HSTA00D (Human TNF-α Quantikine HS ELISA). Intact parathyroid hormone and 25(OH)D levels were measured by electrochemiluminescence and chemiluminescence immunoassays using a Cobas E801 analyzer (Roche Diagnostics GmbH, Mannheim, Germany). Serum total IS levels were measured using a high-performance liquid chromatography-fluorescence detector (HPLC-FLD, Agilent 1100 series; Agilent Technologies, Santa Clara, CA, USA). Serum hemoglobin, total protein, albumin, blood urea nitrogen, creatinine, CRP, and glycated hemoglobin levels were analyzed at each center.

### 5.3. Body Composition and Muscle Strength Measurements

Comorbidities were evaluated using the modified Charlson comorbidity index (CCI) score [23]. Body composition and muscle strength were measured at baseline, as previously described [20,21]. Briefly, body composition was measured using a multifrequency bioimpedance analysis machine (InBody S10, Seoul, Korea) in the supine position. The SMI (kg/m^2^) was calculated as the appendicular muscle mass per height squared. HGS (kg) was measured using a digital dynamometer (Takei 5401; Takei Scientific Instruments Co., Ltd., Niigata, Japan) in the standing position. The participants repeated the experiment thrice with their dominant hand, and the highest value was used in the analysis. Dynamic gait speed (GS) was measured for 6 m with a dynamic start. The participants repeated each type of walking speed test twice, and the mean data were analyzed.

### 5.4. Definition of Sarcopenia and Myostatin/ASM Ratio

Sarcopenia was assessed using the 2019 Asian Working Group for Sarcopenia criteria [24]. Patients with both low muscle mass (SMI < 7.0 kg/m^2^ for men and <5.7 kg/m^2^ for women) and low muscle strength (HGS < 28 kg for men and <18 kg for women) or low physical performance (6 m GS test scores < 1.0 m/s in men and women) were classified as having sarcopenia. Low muscle mass without any decrease in muscle strength or physical performance was classified as presarcopenia.

The myostatin/ASM ratio was defined as the relative serum myostatin level compared to skeletal muscle mass and was calculated as the serum myostatin level (ng/mL) divided by the ASM (kg).

### 5.5. Statistics

The data are presented as the mean ± standard deviation for continuous variables with a normal distribution and as the medians (25th and 75th percentiles) for continuous variables with a non-normal distribution. Categorical variables are presented as number (percentage). Characteristics were analyzed using the independent t-test for continuous variables and the chi-squared test for categorical variables. The correlation between two continuous variables was assessed using Pearson’s correlation analysis. Correction for multiple testing was based on the Bonferroni method. Univariate and multivariate linear regression analyses were performed to examine the relationship between myostatin levels and other variables. Logistic regression analysis was performed to identify factors associated with sarcopenia and presarcopenia. To estimate IS levels as a predictive tool for sarcopenia and presarcopenia, we calculated the AUC value using ROC analysis. All analyses were performed using IBM SPSS Statistics, version 20 (SPSS Inc., Chicago, IL, USA). Statistical significance was set at *p* < 0.05.

## Figures and Tables

**Table 1:** Comparison of clinical characteristics in accordance with myostatin levels.

Characteristics	Myostatin < 4.5 (pg/mL) (n = 76)	Myostatin ≥ 4.5 (pg/mL) (n = 73)	*p* Value
Age (years)	67.0 ± 9.2	63.0 ± 11.9	0.022
Male, n (%)	39 (51.3)	57 (78.1)	0.001
Diabetes mellitus, n (%)	37 (48.7)	38 (52.1)	0.681
Modified CCI score	4.0 (3.0, 5.0)	4.0 (2.0, 5.0)	0.479
Body mass index (kg/m^2^)	24.7 ± 2.8	25.8 ± 3.2	0.030
Laboratory results			
Hemoglobin (g/dL)	12.1 ± 2.1	12.6 ± 2.0	0.103
Total protein (g/dL)	7.0 ± 0.4	6.9 ± 0.5	0.365
Albumin (g/dL)	4.3 (4.1, 4.6)	4.3 (4.2, 4.5)	0.978
Blood urea nitrogen (mg/dL)	31.1 ± 11.9	32.8 ± 10.1	0.348
Creatinine (mg/dL)	1.7 (1.5, 2.2)	2.1 (1.6, 2.5)	0.048
eGFR (mL/min/1.73 m^2^)	34.8 ± 12.7	33.0 ± 12.1	0.395
C-reactive protein (mg/dL)	0.1 (0.0, 0.5)	0.1 (0.0, 0.2)	0.314
TNFa (pg/mL)	1.5 (1.3, 2.1)	1.6 (1.4, 1.9)	0.271
IL-6 (pg/mL)	1.9 (1.2, 2.8)	1.8 (1.2, 3.0)	0.986
iPTH (pg/mL)	67.3 (46.9, 109.0)	68.6 (43.3, 97.8))	0.184
25(OH)D (ng/mL)	14.3 (10.2, 20.3)	14.0 (11.1, 18.4)	0.454
Hemoglobin A1c (%)	6.3 (5.9, 7.3)	6.3 (5.9, 7.5)	0.991
Indoxyl sulfate (mg/dL)	0.4 (0.2, 0.6)	0.3 (0.2, 0.6)	0.141
Myostatin (pg/mL)	3.3 ± 0.8	6.4 ± 1.7	<0.001
Myostatin/SMI	0.5 ± 0.1	0.8 ± 0.2	<0.001
Muscle function			
SMI (kg/m^2^)	7.3 ± 1.2	8.1 ± 1.1	<0.001
HGS (kg)	26.2 ± 9.6	30.6 ± 7.7	0.003
6 m GS (m/s)	1.1 (0.9, 1.3)	1.1 (0.8, 1.4)	0.580
presarcopenia, n (%)	13 (17.1)	4 (5.6)	0.028
low muscle strength, n (%)	24 (31.6)	15 (20.5)	0.126
low physical performance, n (%)	34 (44.7)	31 (42.5)	0.780
Sarcopenia, n (%)	11 (14.5)	3 (4.2)	0.032

Data are expressed as means ± standard deviations, medians (25th and 75th percentiles), or number (percentage). Abbreviations: CCI, Charlson comorbidity index; eGFR, estimated glomerular filtration rate; TNFα, tumor necrosis factor α; IL-6, interleukin-6; iPTH, intact parathyroid; 25(OH)D, 25-hydroxyvitamin D; SMI, skeletal muscle mass index; HGS, handgrip strength; GS, gait speed.

**Table 2:** Correlation between indoxyl sulfate and myostatin and variables.

Characteristics	Indoxyl Sulfate (n = 150)	*p* Value	Myostatin (n = 149)	*p* Value
Age (years)	0.136	0.491	−0.226	0.016
Modified CCI score	0.136	0.292	−0.096	0.738
Body mass index (kg/m^2^)	−0.169	0.119	0.147	0.221
Laboratory results				
Hemoglobin (g/dL)	−0.402	<0.001	0.158	0.165
Total protein (g/dL)	0.080	0.989	−0.055	1.000
Albumin (g/dL)	0.009	1.000	−0.055	1.000
Blood urea nitrogen (mg/dL)	0.478	<0.001	0.007	1.000
Creatinine (mg/dL)	0.636	<0.001	0.137	0.284
eGFR (mL/min/1.73 m^2^)	−0.612	<0.001	−0.073	1.000
C-reactive protein (mg/dL)	0.079	1.000	−0.109	0.727
TNFa (pg/mL)	0.051	1.000	−0.041	1.000
IL-6 (pg/mL)	0.211	0.030	−0.046	1.000
iPTH (pg/mL)	0.433	<0.001	−0.071	1.000
25(OH)D (ng/mL)	−0.141	0.253	−0.023	1.000
Hemoglobin A1c (%)	0.033	1.000	0.074	1.000
Indoxyl sulfate (mg/dL)			−0.237	0.011
Myostatin (pg/mL)	−0.237	0.011		
Myostatin/SMI	−0.141	0.261	0.908	<0.001
Muscle function				
SMI (kg/m^2^)	−0.309	<0.001	0.382	<0.001
HGS (kg)	−0.210	0.029	0.274	0.002
6 m GS (m/s)	−0.087	0.870	0.085	0.906

Abbreviations: CCI, Charlson comorbidity index; eGFR, estimated glomerular filtration rate; TNFα, tumor necrosis factor α; IL-6, interleukin-6; iPTH, intact parathyroid; 25(OH)D, 25-hydroxyvitamin D; SMI, skeletal muscle mass index; HGS, handgrip strength; GS, gait speed.

**Table 3:** Comparison of clinical characteristics in accordance with indoxyl sulfate levels.

Characteristics	IS < 0.365 mg/dL (n = 75)	IS ≥ 0.365 mg/dL (n = 75)	*p* Value
Age (years)	64.9 ± 10.6	65.2 ± 11.0	0.856
Male, n (%)	58 (77.3)	39 (52.0)	0.001
Diabetes mellitus, n (%)	37 (49.3)	39 (52.0)	0.744
Modified CCI score	4.0 (3.0, 5.0)	4.0 (3.0, 5.0)	0.389
Body mass index (kg/m^2^)	25.6 ± 3.0	24.9 ± 3.1	0.217
Laboratory results			
Hemoglobin (g/dL)	13.3 ± 2.1	11.4 ± 1.5	<0.001
Total protein (g/dL)	6.9 ± 0.5	7.0 ± 0.5	0.660
Albumin (g/dL)	4.3 (4.2, 4.5)	4.3 (4.1, 4.5)	0.467
Blood urea nitrogen (mg/dL)	27.4 ± 9.2	36.5 ± 11.0	<0.001
Creatinine (mg/dL)	1.6 (1.5, 2.0)	2.2 (1.7, 2.9)	<0.001
eGFR (mL/min/1.73 m^2^)	40.1 ± 10.8	27.6 ± 10.8	<0.001
C-reactive protein (mg/dL)	0.1 (0.0, 0.4)	0.1 (0.0, 0.3)	0.251
TNFa (pg/mL)	1.4 (1.1, 1.8)	1.8 (1.5, 2.1)	0.910
IL-6 (pg/mL)	1.7 (1.2, 2.6)	2.2 (1.4, 3.2)	0.019
iPTH (pg/mL)	51.7 (37.8, 78.9)	83.5 (60.1, 125.4)	0.001
25(OH)D (ng/mL)	14.7 (11.6, 21.2)	13.4 (10.0, 15.9)	0.016
Hemoglobin A1c (%)	6.2 (5.9, 7.1)	6.4 (5.9, 7.5)	0.116
Indoxyl sulfate (mg/dL)	0.2 (0.2, 0.3)	0.6 (0.4, 1.0)	<0.001
Myostatin (pg/mL)	5.1 ± 2.1	4.5 ± 1.9	0.070
Myostatin/SMI	0.6 ± 0.2	0.6 ± 0.2	0.925
Muscle function			
SMI (kg/m^2^)	8.2 ± 1.1	7.3 ± 1.2	<0.001
HGS (kg)	30.9 ± 9.2	26.0 ± 8.2	0.001
6 m GS (m/s)	1.1 (0.8, 1.3)	1.1 (0.8, 1.4)	0.414
presarcopenia, n (%)	4 (5.4)	13 (17.3)	0.022
low muscle strength, n (%)	14 (18.7)	25 (33.3)	0.041
low physical performance, n (%)	35 (46.7)	30 (40.0)	0.410
Sarcopenia, n (%)	3 (4.1)	11 (14.7)	0.026

Data are expressed as means ± standard deviations, medians (25th and 75th percentiles), or number (percentage). Abbreviations: CCI, Charlson comorbidity index; eGFR, estimated glomerular filtration rate; TNFα, tumor necrosis factor α; IL-6, interleukin-6; iPTH, intact parathyroid; 25(OH)D, 25-hydroxyvitamin D; SMI, skeletal muscle mass index; HGS, handgrip strength; GS, gait speed.

**Table 4:** Independent factors associated with presarcopenia and sarcopenia.

	Presarcopenia		Sarcopenia	
	HR ^a^ (95% CI)	*p* Value	HR ^a^ (95% CI)	*p* Value
Age (years)	1.10 (1.02–1.18)	0.011	1.12 (1.03–1.22)	0.009
Male, n (%)	2.21 (0.69–7.11)	0.183	2.94 (0.80–10.77)	0.104
Diabetes mellitus, n (%)	1.30 (0.40–4.20)	0.660	1.85 (0.49–6.98)	0.362
Creatinine (mg/dL)	0.64 (0.20–2.02)	0.449	0.61 (0.17–2.17)	0.442
Myostatin/SMI	4.09 (0.22–75.57)	0.344	5.01 (0.19–135.76)	0.338
Indoxyl sulfate (mg/dL)	6.25 (1.14–34.23)	0.035	6.67 (1.08–41.44)	0.042

^a^ Clinical parameters (age, gender, diabetes mellitus, creatinine, myostatin/SMI, and indoxyl sulfate) were examined with presence of presarcopenia and sarcopenia (n = 149). Abbreviations: SMI, skeletal muscle mass index; IS, indoxyl sulfate; HR, hazard ratio; CI, confidence interval.

## Data Availability

Data are available in a publicly accessible repository.

## Supplementary Materials

The following supporting information can be downloaded at https://www.mdpi.com/article/10.3390/toxins14100660/s1, Table S1: Baseline characteristics of the study; Table S2: Baseline characteristics according to CKD stages; Table S3. Associations of myostatin levels with variables by linear regression analyses.
