# Supplementary material for: Indoxyl Sulfate Might Play a Role in Sarcopenia, While Myostatin Is an Indicator of Muscle Mass in Patients with Chronic Kidney Disease: Analysis from the RECOVERY Study
Source: Toxins (Basel). 2022 Sep 23;14(10):660. doi: 10.3390/toxins14100660 (PMC9610577; doi:10.3390/toxins14100660)
Supplement: Supplementary file 1 [file toxins-14-00660-s001.zip › toxins-1901320-supplementary.pdf]

toxins

Table S1. Baseline characteristics of the study.

Characteristics	Total (n = 150)	Female (n = 53)	Male (n = 97)	P value
Age (years)	65.0 ± 10.8	63.9 ± 11.4	65.6 ± 10.4	0.347
Male, n (%)	97 (64.7)	24 (45.3)	52 (53.6)	0.330
Diabetes mellitus, n (%)	76 (50.7)	4.0 (3.0, 5.0)	4.0 (2.5, 5.0)	0.406
Modified CCI score	4.0 (3.0, 5.0)	25.2±3.6	25.3 ± 2.7	0.843
Body mass index (kg/m^2)	25.3 ± 3.0			
Laboratory results				
Hemoglobin (g/dL)	12.3 ± 2.1	11.2 ± 1.5	12.9 ± 2.1	<0.001
Total protein (g/L)	7.4 ± 5.3	7.0 ± 0.5	6.9±0.5	0.165
Albumin (g/L)	4.3 (4.1, 4.5)	4.3 (4.0, 4.5)	4.3 (4.2, 4.6)	0.576
Blood urea nitrogen (mg/dL)	32.0 ± 11.0	34.0 ± 12.5	30.8 ± 10.0	0.089
Creatinine (mg/dL)	1.9 (1.6, 2.5)	1.9 (1.6, 2.5)	1.9 (1.6, 2.4)	0.560
eGFR (mL/min/1.73 m^2)	33.8 ± 12.5	29.3 ± 11.7	36.3 ± 12.2	0.001
C-reactive protein (mg/dL)	0.1 (0.0, 0.3)	0.1 (0.0, 0.3)	0.1 (0.0, 0.4)	0.089
TNFa (pg/mL)	1.6 (1.3, 2.0)	1.7 (1.4, 2.2)	1.5 (1.3, 1.9)	0.530
IL-6 (pg/mL)	1.8 (1.3, 2.9)	2.0 (1.3, 3.4)	1.8 (1.2, 2.9)	0.443
iPTH (pg/mL)	68.1 (45.7, 101.9)	81.7 (57.3, 130.2)	64.3 (39.0, 90.3)	0.013
25(OH)D (ng/mL)	14.3 (10.7, 18.6)	11.9 (9.5, 16.9_	14.8 (11.8, 20.1)	0.141
Hemoglobin A1c (%)	6.3 (5.9, 7.4)	6.4 (5.9, 7.5)	6.3 (5.9, 7.3)	0.143
Indoxyl sulfate (pg/mL)	0.4 (0.2, 0.6)	0.5 (0.3, 0.9)	0.3 (0.2, 0.5)	0.044
Myostatin (pg/mL)	4.8 ± 2.0	4.3±2.2	5.1±1.9	0.018
Myostatin/SMI	0.6 ± 0.2	0.6±0.2	0.6±0.2	0.798
Muscle function				
SMI (kg/m^2)	7.7 ± 1.2	6.8 ± 1.1	8.2 ± 1.0	<0.001
HGS (kg)	28.4 ± 9.0	20.4 ± 5.0	32.9 ± 7.6	<0.001
6-m GS (m/s)	1.1 (0.8, 1.3)	1.0 (0.8, 1.2)	1.1 (0.8, 1.3)	0.158
presarcopenia, n (%)	17 (11.4)	9 (17.3)	8 (8.2)	0.097
low muscle strength, n (%)	39 (26.0)	16 (30.2)	23 (23.7)	0.387
low physical performance, n (%)	65 (43.3)	28 (52.8)	37 (38.1)	0.083
Sarcopenia, n (%)	14 (9.3)	8 (15.4)	6 (6.2)	0.067

Data are expressed as means ± standard deviations, medians (25 소, 75 소 percentiles), or number (percentage). Abbreviations: CCI, Charlson comorbidity index; eGFR, estimated glomerular filtration rate; TNFα, tumor necrosis factor α; IL-6, interleukin-6; iPTH, intact parathyroid; 25(OH)D, 25-hydroxyvitamin D; SMI, skeletal muscle mass index; HGS, handgrip strength; GS, gait speed.

Table S2. Baseline characteristics according to CKD stages.

Characteristics	Stage3A (n = 37)	Stage3B (n = 51)	Stage4 (n = 54)	Stage5 (n = 8)
Age (years)	63.7 ± 8.8	65.7 ± 9.9	64.6 ± 12.9	70.0 ± 7.4
Male, n (%)	30 (81.1)	36 (70.6)	28 (51.9)	3 (37.5)
Diabetes mellitus, n (%)	15 (40.5)	24 (47.1)	33 (61.1)	4 (50.0)
Modified CCI score	3.0 (2.0, 5.0)	4.0 (2.0, 5.0)	4.0 (3.0, 6.0)	5.0 (2.5, 6.0)
Body mass index (kg/m^2)	25.5 ± 2.5	25.4 ± 3.0	25.3 ± 3.4	22.5 ± 2.9
Laboratory results				
Hemoglobin (g/dL)	13.8 ± 2.0	12.4 ± 1.6[a]	11.5 ± 1.9[a]	10.8 ± 1.6[a]
Total protein (g/L)	7.0 ± 0.5	6.9 ± 0.4	6.9 ± 0.5	7.2 ± 0.5
Albumin (g/L)	4.4 (4.3, 4.7)	4.3 (4.2, 4.5)	4.3 (3.9, 4.5)[a]	4.4 (4.0, 4.7)
Blood urea nitrogen (mg/dL)	23.6 ± 6.3	29.4 ± 8.4[a]	37.5 ± 9.8[ab]	50.3 ± 11.2[abc]
Creatinine (mg/dL)	1.5 (1.3, 1.6)	1.8 (1.6, 2.0)[a]	2.5 (2.1, 2.9)[ab]	3.7 (3.4, 4.5)[abc]
eGFR (mL/min/1.73 m^2)	50.7 ± 4.8	36.3 ± 3.9[a]	23.0 ± 4.1[ab]	13.0 ± 1.4[abc]
C-reactive protein (mg/dL)	0.1 (0.0, 0.4)	0.1 (0.1, 0.5)	0.1 (0.0, 0.2)	0.1 (0.0, 0.6)
TNFa (pg/mL)	1.3 (1.0, 1.7)	1.5 (1.2, 1.7)	1.8 (1.5, 2.1)	2.0 (1.9, 2.5)
IL-6 (pg/mL)	1.5 (1.0, 2.4)	1.7 (1.2, 2.4)	2.7 (1.5, 3.6)	2.6 (1.6, 3.6)
iPTH (pg/mL)	48.1 (33.3, 65.1)	59.0 (42.7, 75.8)	92.1 (69.9, 151.1)[ab]	149.0 (109.1, 191.1)[abc]
25(OH)D (ng/mL)	14.6 (11.9, 19.7)	15.3 (11.5, 23.2)	12.5 (9.6, 15.5)	13.4 (10.9, 18.4)
Hemoglobin A1c (%)	6.0 (5.5, 6.6)	6.6 (5.9, 7.5)	6.5 (6.0, 7.9)[a]	6.3 (5.7, 6.8)
Indoxyl sulfate (pg/mL)	0.2 (0.1, 0.3)	0.3 (0.2, 0.4)	0.6 (0.3, 0.9)[ab]	1.2 (1.0, 1.6)[abc]
Myostatin (pg/mL)	4.5 ± 1.5	4.7 ± 1.6	5.2 ± 2.5	3.9 ± 2.6
Myostatin/SMI	0.6 ± 0.2	0.6 ± 0.2	0.7 ± 0.3	0.6 ± 0.3
Muscle function				
SMI (kg/m^2)	8.0 ± 1.1	7.9 ± 1.1	7.5 ± 1.3	6.4 ± 0.9[ab]
HGS (kg)	31.9 ± 8.8	28.9 ± 9.5	26.6 ± 8.3	21.7 ± 4.7[a]
6-m GS (m/s)	1.0 (0.8, 1.2)	1.0 (0.9, 1.3)	1.1 (0.8, 1.3)	1.0 (0.8, 1.2)
presarcopenia, n (%)	2 (5.4)	6 (11.8)	5 (9.4)	4 (50.0)
low muscle strength, n (%)	6 (16.2)	14 (27.5)	16 (29.6)	3 (37.5)
low physical performance, n (%)	18 (48.6)	21 (41.2)	22 (40.7)	4 (50.0)
Sarcopenia, n (%)	2 (5.4)	4 (7.8)	4 (7.5)	4 (50.0)

Statistical significances were tested by Oneway analysis of variances among groups. [a]*P* value < 0.05 (mean values are significantly different from the Stage 3A group). [b]*P* value < 0.05 (mean values are significantly different from the Stage 3B group). [c]*P* value < 0.05 (mean values are significantly different from the Stage 4 group).

Table S3. Associations of myostatin levels with variables by linear regression analyses.

	Univariate		Multivariate	
**Characteristics**	**β±SE**	**P value**	**β±SE**	**P value**
Age (years)	-0.043 ± 0.015	0.005	-0.020 ± 0.013	0.114
Modified CCI score	-0.102 ± 0.088	0.246		
Body mass index (kg/m^2)	0.091 ± 0.051	0.074		
Laboratory results				
Hemoglobin (g/dL)	0.155 ± 0.080	0.055		
Total protein (g/dL)	-0.236 ± 0.354	0.505		
Albumin (g/dL)	-0.321 ± 0.484	0.508		
Blood urea nitrogen (mg/dL)	0.001 ± 0.015	0.928		
Creatinine (mg/dL)	0.384 ± 0.228	0.095	1.134 ± 0.245	<0.001
eGFR (mL/min/1.73 m^2)	-0.012 ± 0.013	0.377		
C-reactive protein (mg/dL)	-0.173 ± 0.147	0.242		
TNFa (pg/mL)	-0.052 ± 0.104	0.619		
IL-6 (pg/mL)	-0.053 ± 0.096	0.581		
iPTH (pg/mL)	-0.002 ± 0.002	0.392		
25(OH)D (ng/mL)	-0.005 ± 0.019	0.782		
Hemoglobin A1c (%)	0.120 ± 0.152	0.431		
Indoxyl sulfate (pg/mL)	-1.147 ± 0.387	0.004	-1.853 ± 0.441	<0.001
Muscle function				
SMI (kg/m^2)	0.590 ± 0.118	<0.001	0.415 ± 0.144	0.005
HGS (kg)	0.062 ± 0.018	0.001	0.007 ± 0.019	0.704
6-m GS (m/s)	0.579 ± 0.559	0.302		

Abbreviations: CCI, Charlson comorbidity index; eGFR, estimated glomerular filtration rate; TNFα, tumor necrosis factor α; IL-6, interleukin-6; iPTH, intact parathyroid; 25(OH)D, 25-hydroxyvitamin D; SMI, skeletal muscle mass index; HGS, handgrip strength; GS, gait speed.
